# Breastfeeding Practices and Postpartum Depression in Mexican Women during the COVID-19 Pandemic: A Cross-Sectional Study

**DOI:** 10.3390/medicina59071330

**Published:** 2023-07-19

**Authors:** Mariana Chávez-Tostado, Karla Verónica Chávez-Tostado, Gabino Cervantes-Guevara, Guillermo Cervantes-Cardona, Diana Mercedes Hernandez-Corona, Tonatiuh González-Heredia, Miriam Méndez-del Villar, Fernanda Isadora Corona-Meraz, Milton Omar Guzmán-Ornelas, Francisco José Barbosa-Camacho, Andrea Socorro Álvarez-Villaseñor, Enrique Cervantes-Pérez, Clotilde Fuentes-Orozco, Natalia Guadalupe Barrera-López, Noelia Esthela López-Bernal, Alejandro González-Ojeda

**Affiliations:** 1 Departamento de Reproducción, Centro Universitario de Ciencias de la Salud, Universidad de Guadalajara, Guadalajara 44410, Mexico; 2 Departamento de Cirugía General y Endócrina, Hospital General Medio Ajusco, Ciudad de México 04510, Mexico; 3 Departamento de Bienestar y Desarrollo Sustentable, Centro Universitario del Norte, Universidad de Guadalajara, Colotlán 46200, Mexico; 4 Departamento de Disciplinas Filosóficas, Metodológicas e Instrumentales, Centro Universitario de Ciencias de la Salud, Universidad de Guadalajara, Guadalajara 44410, Mexico; 5 Departamento de Ciencias Biomédicas, División de Ciencias de la Salud, Centro Universitario de Tonalá, Universidad de Guadalajara, Tonalá 45425, Mexico; 6 Departamento de Psiquiatría, Hospital Civil Fray Antonio Alcalde, Universidad de Guadalajara, Guadalajara 44340, Mexico; 7 Coordinación Auxiliar Médica de Investigación en Salud, Instituto Mexicano del Seguro Social, La Paz 23060, Mexico; 8 Departamento de Medicina Interna, Hospital Civil de Guadalajara “Fray Antonio Alcalde”, Universidad de Guadalajara, Guadalajara 44340, Mexico; 9 Unidad de Investigación Biomédica 02, Hospital de Especialidades, Centro Médico Nacional de Occidente, Instituto Mexicano del Seguro Social, Guadalajara 44340, Mexico

**Keywords:** COVID-19, postpartum depression, breastfeeding, pandemic, skin-to-skin contact

## Abstract

*Background*: Breastfeeding is a characteristic process of mammals that ensures delivery of an adequate nutritional supply to infants. It is the gold standard food source during an infant’s first months of life. Since the onset of the COVID-19 pandemic in 2020, people in quarantine have experienced a wide range of feelings, which may make isolation challenging in terms of maternal health. This study focused on the prevalence of breastfeeding practices and postpartum depression (PPD) among Mexican women during the COVID-19 pandemic. *Materials and Methods*: This cross-sectional study included 586 postpartum women who completed an online survey 4−8 weeks after delivery from April to December 2020 in Guadalajara, Mexico. The aim was to identify potentially depressed mothers according to the Edinburgh Postnatal Depression Scale (EPDS) and describe their breastfeeding practices. *Results*: The mean maternal age was 30.4 ± 4.6 years, the mean EPDS score was 9.6 ± 5.0, and the PPD prevalence according EPDS scores was 27.1%. Exclusive breastfeeding (EBF) was reported by 32.3% of mothers in the first 48 h and by 70.3% of mothers 48 h after delivery. EBF was associated with a lower prevalence of PPD during the first 48 h (*p* = 0.015) and after the first 48 h (*p* = 0.001) after delivery. Skin-to-skin contact (SSC) was reported by 385 (65.7%) mothers. PPD was less frequent in mothers practicing SSC (20.3%) than it was in those not practicing SSC (40.3%) (*p* = 0.001). A higher percentage of mothers practiced SSC breastfed (66.9%) and used EBF (150, 79.4%) (*p* = 0.012 and 0.001, respectively). *Conclusions*: Results suggest that the pandemic emergency and restrictions imposed on the population significantly affected the well-being of mothers after birth, and that these effects may have posed risks to the mental health and emotional stability of postpartum mothers. Therefore, encouraging BF or EBF and SSC may improve or limit depressive symptoms in postpartum mothers.

## 1. Introduction

The COVID-19 pandemic was an unprecedented global crisis that challenged the approach to almost every aspect of life [1]. Since the onset of the pandemic, over 691,207,603 cases of COVID-19 and 6,898,266 deaths have occurred globally [2]. Many countries, including Mexico, endured prolonged lockdown measures to encourage social distancing and limit the spread of the virus [3]. By the end of 2020, a total of 205 peripartum mothers died, equivalent to a fatality rate of 1.93% and a maternal mortality rate of 10.1 per 100,000 live births [4].

Breastfeeding (BF) is the gold standard food source during an infant’s first months of life; the World Health Organization and the United Nations International Children’s Emergency Fund recommend exclusive breastfeeding for at least the first 6 months of life [5]. Despite solid evidence of the nutritional and immunological benefits of early breastfeeding in reducing neonatal mortality and morbidity [6], only 50% of newborns in the world are breastfed during their first hour [7].

Depression is a very common psychological disorder, especially in women in the postpartum period; it affects 17.22% of the world’s population. Prevalence rates range from 0.5% to approximately 60%, depending on cultural variations and practices in different countries [8]. Postpartum depression manifests secondary to hormonal changes and fatigue after birth [9]. It is a severe psychiatric disease that is underdiagnosed and understudied (both clinically and experimentally).

Approximately 20% of postpartum deaths in 2020 were due to suicide, making it the most common birth complication that hurts the mother [10].

People in quarantine experience a wide range of feelings, which may make isolation challenging in terms of maternal health [11,12]. The COVID-19 pandemic has had an impact on the rate of postpartum depression (PPD) and BF in postpartum mothers [13]. PPD occurs mainly within 4 to 6 weeks after childbirth, may continue for up to 1 year [14], and can affect lactogenesis and BF after childbirth [15,16]. The symptoms of PPD are similar to those of major depressive disorders [17,18]. In addition, women with PPD also experience guilt about their inability to care for their newborn baby [19].

According to some studies, the COVID-19 pandemic has been associated with an increased risk of mental health problems in pregnant and postpartum women [20].

Information regarding the prevalence of mental health problems and BF in postpartum Mexican women during the COVID-19 pandemic is scarce. Access to information regarding the increase in the incidence of PPD during the pandemic can be used as a reference for decisions and policies of governments’ health systems. This study investigated the effects of the COVID-19 pandemic on the prevalence of PPD and BF practices in postpartum Mexican women.

## 2. Materials and Methods

### 2.1. Study Setting

This was a cross-sectional study that included an online survey to identify mothers with potential PPD according to the Edinburgh Postnatal Depression Scale (EPDS), with validation in puerperal women in Mexico [21] during the COVID-19 pandemic from April to December 2020 in Guadalajara, Mexico. This manuscript was prepared following STROBE guidelines for observational studies [22].

After delivery, all participants were invited to complete an online survey at 4−8 weeks postpartum; mothers who agreed to participate gave their informed consent. The online survey was sent by email to participants. Patients’ names, contact information, and locations were not requested. Those who agreed to participate did so through the survey.

The study protocol followed the Declaration of Helsinki Ethical Principles for Medical Research Involving Human Subjects, and the study was approved by the Research Ethics Committee of the “Civil Hospital Fray Antonio Alcalde” (122/20), and with the clinical trial number NCT04769700 (ClinicalTrials.gov). A preliminary report regarding design, methods, and partial results was prepared for our group. This article constitutes the final information and results of the protocol [23].

### 2.2. Instruments

PPD was evaluated using the EPDS scale validated using the Spanish version for Mexican postpartum women [21,24]. This instrument is a self-administered questionnaire consisting of 10 items designed to detect PPD symptoms using a 0 to 3 point scale according the response given by mothers how they have felt in the last seven days, and dealing with the ability to laugh, sleep, pleasure, guilt, anxiety, fear, overwhelm, sadness, crying, and self-injury. The cutoff point for the risk of PPD was set at 13 points [25]. The online survey was developed by the research team, whose members have expertise in the academic and research fields of dietetics, child nutrition, and pediatric clinical care.

### 2.3. Participants

Demographic and clinical characteristics were obtained from the participants’ clinical records. Inclusion criteria included women aged ≥18 years with a single birth, 4–8 weeks postpartum, and having the ability to answer the online survey. Exclusion criteria were incomplete surveys, stillbirth, or a previous psychiatric disorder.

Data collected included sociodemographic characteristics (maternal age and education level), parity, mode of delivery, occupation, anthropometric data, previous lactation history, and the use of exclusive breastfeeding (EBF) or combined BF during the first 48 h and after 48 h after giving birth. The practice of skin-to-skin contact (SSC) was also recorded.

### 2.4. Statistical Analysis

Results are expressed as mean and standard deviation or number and percentage. IBM SPSS Statistics (version 21; IBM Corp., Armonk, NY, USA) was used for statistical analysis. Categorical variables are expressed as percentage and raw number, and continuous variables are expressed as the mean ± standard deviation. Data were analyzed using the Student’s *t*-test or the nonparametric Mann−Whitney *U* test for quantitative data and the χ^2^ test or Fisher’s exact test for qualitative data. Differences were considered significant at *p* < 0.05.

## 3. Results

A total of 586 postpartum mothers completed the questionnaire. Their mean age was 30.4 ± 4.6 years. The mean gestational age at the time of birth was 38.9 ± 0.9 weeks. Vaginal delivery occurred in 451 cases (77.7%), while 135 cases (23%) resulted in cesarean section. Of the mothers, 356 were primiparous (67.6%). The weight of the newborns was 3.3 ± 1.4 kg. Using the cutoff for PPD as an EPDS score of 13, 159 mothers (27.1%) were identified as having PPD; the global average EPDS score was 9.6 ± 5.02 (Table 1).

During the first 48 h after delivery, 189 mothers (32.3%) used EBF. The rates of PPD were 24.5% among mothers who reported EBF and 30.2% in those who did not report EBF (*p* = 0.015; odds ratio (OR) 95%; confidence interval (CI) 1.4 (1.06–2.01)). After the first 48 h after delivery, PPD was reported by 23.3% of mothers who practiced EBF and by 36.2% of mothers who did not (*p* = 0.001; OR 1.5 (1.1–2.02)).

SSC was reported by 385 mothers (65.7%). PPD was less frequent in mothers who used SSC (20.3%) than it was in those who did not use SSC (40.3%) (*p* = 0.001; OR 95%; CI 1.9 (1.5–2.5)). The presence of PPD was also less frequent in mothers who were assessed for lactation counseling (24%) than in those who were not (32.3%) (*p* = 0.028; OR 95%; CI 1.3 (1.03–1.7)). Detailed results are shown in Table 2.

During the first 48 h after birth, mothers who practiced SSC had a higher frequency of BF (370, 66.9%) and EBF (150, 79.4%) (*p* = 0.012; OR 95%; CI 1.6 (1.1–2.3) and *p* = 0.001; OR 95%; IC 1.9 (1.4–2.6), respectively). BF counseling was reported by 363 (61.9%) mothers, and 264 (72.7%) mothers used SSC (*p* = 0.00; OR 95%; CI 1.6 (1.3–2.0)). Detailed results are shown in Table 3.

## 4. Discussion

This cross-sectional study’s purpose was to identify an association between BF practices and PPD in Mexican postpartum mothers during the COVID-19 pandemic. We found that a high percentage of our cohort (27.1%) reported PPD, as identified using the EPDS, during the COVID-19 pandemic. This frequency was significantly higher than that reported before the COVID-19 pandemic in Mexico (13.3–18%) [26]. An increase in the prevalence of PPD has been documented internationally since the onset of the COVID-19 pandemic. The annual frequency of PPD before the COVID-19 pandemic was reported as 6.9–12.9% in high-income countries and 20% in low- and middle-income countries [27,28].

The prevalence of PPD has been reported as 30% in China [29], 40.7% in Canada [30], 34% in Turkey [13], 32.8–47.5% in the United Kingdom [31], 38% in the United State [32], and 39.2% in a recent study in Mexico [33]. These findings suggest that the pandemic and measures adopted to fight its spread may have had negative effects on the psychological well-being of postpartum women [34]. Another study by Yahya et al. [35] found that 27% of mothers in their study had a higher risk of depression based on their EPDS scores (8.14 ± 6.00).

Some studies have reported that mothers with depressive symptoms are more likely to abandon the practice of EBF [36,37]. We observed that the prevalence of EBF during the first 48 h after birth was lower in mothers with PPD (24.5%) than it was in those without PPD (79.4%) (*p* = 0.015). A similar pattern was observed for EBF after 48 h after delivery in mothers with PPD (23.3%) and without PPD (76.7%) (*p* = 0.001). These results are similar to those reported by a cross-sectional study of 1799 postpartum mothers in Europe during the COVID-19 pandemic, which reported a PPD frequency of 17% when assessed using the EPDS. This study also found that one risk factor for major depressive symptoms was not practicing BF (OR 1.86 (1.26–2.74)) [38].

Liu et al., in a cross-sectional study involving 1136 women, reported prevalence rates of PPD and postpartum post-traumatic stress disorder (PP-PTSD) symptoms of 23.5 and 6.1%, respectively, and revealed that the biggest risk factor for PPD symptoms was the existence of PP-PTSD. Low sleep quality, a lack of social support, and infant incubator admission were additional PPD risk factors [39].

Another cross-sectional study conducted in Edirne, Turkey, involving 111 pregnant women in the third trimester, aimed to investigate the prevalence and contributing variables of PPD. In the first month after delivery, PPD occurred in 14% (*n* = 14) of mothers, and in the second month it increased to 17% (*n* = 17). The probability of experiencing PPD, measured using the EPDS, was significantly higher among younger mothers, mothers with unemployed husbands, mothers with lower income, mothers whose child had a health problem, and mothers who did not breastfeed [40].

A cross-sectional study conducted in Sao Paulo with 315 women between the ages of 14 and 44 by Oliveira et al. found that 62% of patients had depression. In the multivariate analysis, depression’s causes and psychological aggression during pregnancy were both highly significant predictors of postpartum depression [41].

A systematic review of studies of a total of 20,225 postpartum women during the COVID-19 pandemic reported a 26.7% prevalence of PPD symptoms; subgroup analyses revealed that postpartum women who did not practice BF experienced a higher risk of depressive symptoms [42].

SSC is an effective method for instigating mother–infant bonding [43], and should be established in the first hours after birth to begin the healthy mothering process [44]. SSC is an effective and simple intervention that reduces the prevalence of PPD symptoms [34,45,46]. The information offered to mothers during the prenatal period may help improve the practice of BF. In our study, 65.7% of postpartum mothers performed BF; 20.3% of mothers who reported SSC had PPD, whereas 40.3% of mothers who did not report SSC had PPD (*p* = 0.001). During the first 48 h after birth, mothers who practiced SSC used EBF more frequently (79.4%) than did those who did not practice SSC (59.2%) (*p* = 0.001). A similar result was observed for BF: 66.9% of mothers who used SSC practiced BF, but only 33.1% of mothers who did not use SSC practiced BF (*p* = 0.001) [47]. These results suggest that SSC may help reduce the risk of PPD by promoting BF and EBF.

In a systematic review by Moore et al. [48], women who initiated SSC also breastfed their infants for longer and were more likely to breastfeed exclusively between the time of hospital discharge and 1 month later, and between 6 weeks and 6 months after birth.

Bigelow et al. [49] indicated that mother–infant SSC provides benefits by decreasing mothers’ depressive symptoms and physiological stress in the first weeks after birth. SSC can also improve general health and reduce symptoms of depression and stress in new mothers [34,45,48,49,50].

Also, we observed significant numbers regarding the performance of cesarean sections in results reported before the COVID-19 pandemic. This might be related to the fact that cesarean section is considered a more convenient mode of delivery by both medical staff and women, compared with vaginal delivery [51,52,53]. Also, this surgical procedure may have increased during COVID-19 because it is believed to be a safer and rapid method of delivery [53]. Moreover, there was an increase in caesarean sections observed in China among COVID-19 infected women [54].

Postpartum depression results in parenting challenges and unfavorable consequences for early childhood development, which in turn have a detrimental impact on a mother’s mental health. Recommendations include a follow-up evaluation for a suspected major depressive condition, as well as a family’s provision of strong social support. As with other psychiatric disorders, a new mother is hesitant to discuss her sad mood or seek assistance. This increases her likelihood of developing a major depressive disorder (MDD) with peripartum origins [55].

It is recommended that government officials, psychologists, and health managers receive training in stress management to detect and diagnose women with a history of mental problems and to implement programs and guidelines for mental health support during and after pregnancy [56].

Our study has some limitations, one of which is that there are currently no articles regarding BF practices associated with PPD in the Mexican population during the period of the COVID-19 pandemic to compare with our results; at the same time, this is what makes our work important, as we report what happened in our environment (Mexico) regarding breastfeeding. Our data provide the basis for a better understanding and practice of breastfeeding. The pandemic was accompanied by a financial crisis and social problems that were not evaluated in our study, but should be considered as possible variables, in addition to the fear of contagion and loss of family members, which could have affected the mental health of mothers.

## 5. Conclusions

Our study aimed to contribute to the early detection of PPD and timely intervention using BF, and to provide information regarding the practice of BF in patients with PPD in Mexico during the COVID-19 pandemic. Our results show the practice of BF decreased due to restrictions imposed on the population and that the incidence of PPD increased considerably; this is an important community problem to address.

## Figures and Tables

**Table 1:** Prevalence of demographic characteristics, EPDS score, and breastfeeding practices.

Characteristics		Value *N* = 586
Demographic data	Age (years)	30.4 ± 4.6
	Gestational weeks	38.9 ± 0.9
	Weight of mothers (kg)	75.9 ± 15.7
	BMI (kg/m2)	28.7 ± 6.01
	Weight of newborns (kg)	3.3 ± 1.4
	Primiparous	396 (67.6)
EPDS score	EPDS score	9.6 ± 5.02
	EPDS score ≥ 13	159 (27.1)
Delivery	Vaginal delivery	451 (77)
	Cesarean section	135 (23)
Marital status	Single	33 (5.6)
	Married	415 (70.8)
	Free union	138 (23.5)
Occupation	Student	18 (3.1)
	Housewife	187 (31.9)
	Employed	354 (60.4)
	Unemployed	27 (4.6)
BF in first 48 h after delivery	Exclusive BF	189 (32.3)
	Combined BF	364 (62.1)
	Formula use	33 (5.6)
BF 48 h after delivery	Exclusive BF	412 (70.3)
	Combined BF	162 (27.6)
	Formula use	12 (2)
Previous BF		196 (33.4)
Lactation BF		363 (61.9)
SSC		385 (65.7)

Values are expressed as mean ± standard deviation or number (percentage). EPDS, Edinburgh Postnatal Depression Scale; BMI, body mass index; BF, breastfeeding; SSC, skin-to-skin contact.

**Table 2:** EPDS scores among postpartum mothers.

Indicator		*N* (%)	EPDS Score < 13, *N* (%)	EPDS Score ≥ 13, *N* (%)	*p* Value	OR (95% CI)
Mode of delivery	Vaginal	451 (77.0)	325 (72.1)	126 (27.9)	0.4	1.14 (0.8–1.4)
	Cesarean	135 (23.0)	102 (75.6)	33 (24.4)		
Primiparous	First newborn	396 (67.6)	285 (72)	111 (28)	0.4	1.1 (0.8–1.05)
	Second or more newborn	190 (32.4)	142 (74.7)	48 (25.3)		
Marital status	Single	33 (5.6)	27 (81.8)	6 (18.2)	**0.04**	–
	Married	415 (70.8)	310 (74.7)	105 (25.3)		
	Free union	138 (23.5)	90 (65.2)	48 (34.8)		
Occupation	Student	18 (3.1)	15 (83.3)	3 (16.7)	**0.001**	–
	Housewife	187 (31.9)	139 (74.3)	48 (25.7)		
	Employed	354 (60.4)	264 (74.6)	90 (25.6)		
	Unemployed	27 (4.6)	9 (33.3)	18 (66.7)		
Previous BF	No	390 (66.6)	276 (70.8)	114 (29.2)	**0.1**	1.2 (0.9–1.7)
	Yes	196 (33.4)	151 (77)	45 (23)		
BF in the first 48 h	No	33 (5.6)	24 (72.7)	9 (27.3)	0.9	1.005 (0.5–1.7)
	Yes	553 (94.4)	403 (72.9)	150 (27.1)		
EBF in the first 48 h	No	397 (67.7)	277 (69.8)	120 (30.2)	**0.01**	1.4 (1.06–2.01)
	Yes	189 (32.3)	150 (79.4)	39 (20.6)		
BF after 48 h	No	12 (2)	9 (75)	3 (25)	0.8	1.08 (0.4–2.9)
	Yes	574 (98)	418 (72.8)	156 (27.2)		
EBF after 48 h	No	174 (29.7)	111 (63.8)	63 (36.2)	**0.001**	1.5 (1.1–2.029
	Yes	412 (70.3)	316 (76.7)	96 (23.3)		
BF counseling	No	223 (38.1)	151 (67.7)	72 (32.3)	**0.02**	1.3 (1.03–1.7)
	Yes	363 (61.9)	276 (76)	87 (24)		
Presence of SSC	No	201 (34.3)	129 (59.7)	81 (40.3)	**0.001**	1.9 (1.5–2.5)
	Yes	385 (65.7)	307 (79.7)	78 (20.3)		

EPDS, Edinburgh Postnatal Depression Scale; OR, odds ratio; CI, confidence interval; BF, breasfeeding; EBF, exclusive breastfeeding.

**Table 3:** SSC practice among postpartum mothers.

Indicator		Total *N* (%)	No SSC, *N* (%)	Yes SSC, *N* (%)	*p* Value	OR (95% CI)
Mode of delivery	Vaginal	451 (77)	147 (32.6)	304 (67.4)	0.11	1.12 (0.9–1.3)
	Cesarean	135 (23)	54 (40)	81 (60)		
Primiparous	First newborn	396 (67.6)	132 (33.3)	264 (66.7)	0.47	1.04 (0.9–1.1)
	Second or later newborn	190 (32.4)	69 (36.3)	121 (63.7)		
Previous BF	No	390 (66.6)	138 (35.4)	252 (64.6)	0.4	1.1 (0.8–1.4)
	Yes	196 (33.4)	63 (32.1)	133 (67.9)		
BF in the first 48 h	No	33 (5.6)	18 (54.5)	15 (45.5)	0.012	1.6 (1.1–2.3)
	Yes	553 (94.4)	183 (33.1)	370 (66.9)		
EBF in the first 48 h	No	397 (67.7)	162 (40.8)	235 (59.2)	0.001	1.9 (1.4–2.6)
	Yes	189 (32.3)	39 (20.6)	150 (79.4)		
BF after 48 h	No	12 (2)	6 (50)	6 (50)	0.24	1.3 (0.7–2.3)
	Yes	574 (98)	195 (34)	379 (66)		
EBF after 48 h	No	174 (29.7)	66 (37.9)	108 (62.1)	0.29	1.15 (0.9–1.4)
	Yes	412 (70.3)	135 (32.8)	277 (67.2)		
Breastfeeding counseling	No	223 (38.1)	102 (45.7)	121 (54.3)	0.001	1.6 (1.3–2.0)
	Yes	363 (61.9)	99 (27.3)	264 (72.7)		

SSC, skin-to-skin contact; OR, odds ratio; CI, confidence interval; BF, breastfeeding; EBF, exclusive breastfeeding.

## Data Availability

Data supporting reported results are available upon email request to the corresponding author.
